# Not all distress is the same: profiles of stress, anxiety, and depression among university students

**DOI:** 10.3389/fpsyg.2026.1834233

**Published:** 2026-05-21

**Authors:** Juan Feng, Kai Ma, Zhihui Pan

**Affiliations:** 1 Mental Health Education Center, Xi'an University of Technology, Xi'an, China; 2 School of Teacher Development, Shaanxi Normal University, Xi'an, China; 3 Student Psychological Development Center, Xi'an Technological University, Xi'an, China

**Keywords:** anxiety, depression, latent profile analysis, stress, university students

## Abstract

**Introduction:**

The psychological distress among university students has become more and more common, but the manifestations at the individual level are very heterogeneous. This study was guided by cognitive appraisal theory, which took a personcentered approach to investigate joint patterns of perceived stress, anxiety, and depression in university students and their relationships with sleep quality.

**Methods:**

A total of 764 undergraduate students from a comprehensive university in central China participated in this study. Latent profile analysis was conducted to classify distinct psychological distress profiles of participants. Demographic characteristics and sleep quality indicators were further analyzed as relevant correlates of the identified profiles.

**Results:**

Three latent profiles included a Resilient profile (41.0%) with low stress appraisals and emotional symptoms, an Uncontrollability-Depressive profile (47.9%) with high uncontrollability appraisals and depressive symptoms, and a Tension-Anxious profile (11.1%) with high tension appraisals and anxiety symptoms. These profiles varied not only in terms of the severity of distress overall, but also in terms of configurational patterns of stress appraisals and emotional reactions. Additional analyses indicated that the quality of sleep strongly predicted membership in a particular profile and functional outcomes related to sleep, and worse sleep always corresponded to higher-risk profiles. On the contrary, there were no independent predictive effects of demographic variables after controlling for the effect of sleep quality.

**Discussion:**

This study emphasizes the heterogeneity of psychological distress among university students and the importance of a profile-based approach by combining cognitive appraisal processes and functional outcomes. The results highlight the configurational heterogeneity of student distress and imply sleep as an effective and cost-effective stratified screening and early intervention tool.

## 1 Introduction

Over the past few years, increasing focus has been placed on the various types of psychological distress among university students. The university phase is a pivotal developmental stage between adolescence and adulthood in which students have to manage academic pressures, social adaptation, and role changes at once, making them especially susceptible to prolonged psychological stress (Stokoe et al., 2024). A large amount of research consistently shows that depression, anxiety, and perceived stress are very common among university students, with findings being highly consistent across different cultural backgrounds and academic fields (Paiva et al., 2025; Estrella-Proaño et al., 2024). Systematic reviews and meta-analyses also provide further evidence indicating that the burden of perceived stress among university students is widespread globally, emphasizing its importance as a cross-cultural public health issue (Fentahun et al., 2025). Notably, psychological distress among university students not only influences their emotional well-being and psychological adjustment but may also exert persistent negative effects on academic performance, self-control, and learning processes (Zhang J. et al., 2024). Taken together, these results show that psychological distress among university students is not a minor phenomenon but rather a core developmental problem with broad impact and possible long-term consequences.

Although overall prevalence rates have been widely reported, there is a growing body of literature that has identified the fact that psychological distress among university students does not exist in one form or another. Although students might be subjected to similar academic and life stressors, they can show significant differences in their emotional reaction, functional impairment, and adaptive consequences (Bonanno, 2004). Fried (2022) suggested that mental health issues must be viewed as complex systems that are made up of several interrelated components, instead of traditional symptom syndromes. In this systems view, depression and anxiety can be seen as dynamic network patterns where the elements of emotion, cognition, and behavior are interlinked. Daily life tracking research also shows that perceived stress, anxiety, and depression can take different cyclical courses with time, instead of following a single developmental pattern across people (Feng et al., 2023). The effects of academic and life stressors on the mental well-being of students may differ significantly depending on the cultural and contextual environment because underlying stressors affect the psychological outcomes mostly by the perceived stress and are further influenced by individual buffering factors like coping styles and emotional stability (Slimmen et al., 2022). Thus, the use of general levels or average associations alone cannot be sufficient to represent the structural heterogeneity of psychological distress at an individual level among university students.

The current literature demonstrates that perceived stress, anxiety, and depression are strongly comorbid in university students and have consistent correlational patterns (Alwhaibi et al., 2023). Cross-cultural data also suggest that depression, anxiety, and stress tend to co-occur or be presented in the form of accompanying symptoms and are related to a variety of sociodemographic features (Ike et al., 2025). This structure of co-occurrence has also been documented numerous times among medical students and health-related discipline students, which implies high contextual generalizability (Fauzi et al., 2021). Nevertheless, strong correlations do not mean psychological homogeneity. People can display significantly different emotional and functional response patterns even when they are subjected to similar amounts of stress. Others might have only temporary disturbance and remain fairly stable in their functioning, but others might pursue more chronic distress pathways, which is why there are several routes of adjustment to adversity (Bonanno, 2004). The phenomenon brings out the necessity to go beyond average relationships between variables and consider joint configurational patterns of various psychological indicators on the individual level.

The cognitive appraisal theory developed by Lazarus offers a critical theoretical perspective on the given heterogeneity. This theory indicates that the experiences of stress are not directly influenced by situations, but they emerge due to subjective appraisals of situational meaning and perceived coping resources (Lazarus and Folkman, 1984; Lin et al., 2023). In this model, primary appraisal is concerned with whether or not a situation is seen as threatening, whereas secondary appraisal is concerned with the assessment of control and capacity to cope. Meta-analytic studies evidence stable and differentiated relations between particular appraisal dimensions and emotional reactions, which gives strong empirical evidence in favor of separating cognitive elements of perceived stress (Yeo and Ong, 2024). Other research has also demonstrated that cognitive regulation mechanisms like positive reappraisal have the ability to change meaning-making and have buffering effects on anxiety and depression (Garland et al., 2009; Kivity and Huppert, 2016). Also, it has been established that negative cognitive processing biases interacting with perceived stress are one of the main psychological processes involved in the formation of depression and anxiety (Miao et al., 2024). Collectively, these results indicate that there can be different pathways connecting specific cognitive aspects of stress with emotional symptoms.

Despite the fact that cognitive appraisal theory focuses on individual variations in stress processing, most of the current empirical research has been based on variable-centered methods, which usually presuppose that people within a population work under similar psychological mechanisms (Mathew and Doorenbos, 2022). Although these methods are effective in establishing general correlations between stress, anxiety, and depression, they cannot be used to determine joint patterns of various psychological indicators at the personal level (Saqr et al., 2024). On the other hand, person-based strategies pay attention to the differences among individuals and are more appropriate to draw the boundaries of heterogeneity of psychological structures (Kennedy et al., 2022). The latent profile analysis (LPA) is one of such techniques that enable the combination of several continuous indicators in one model as well as the determination of internally homogeneous subgroups. This method has been generally viewed as a more suitable statistical paradigm in describing heterogeneous forms of psychological distress (Lubke and Muthén, 2007). Past research has started using LPA to analyze subtypes of stress and emotional reactions and has shown significant relationships with diverse functional outcomes, making it theoretically and practically useful (Luo et al., 2021). In addition, the measurement structure of perceived stress has been studied, indicating that various dimensions can have different psychological implications, offering psychometric evidence of profile identification through the lens of cognitive appraisal (Pedersen et al., 2024). However, in the populations of university students, there is still no systematic research that combines cognitive aspects of perceived stress with symptoms of anxiety and depression in an integrated person-centered framework that is based on the theory of cognitive appraisal.

In the analysis of latent trends of psychological distress in university students, external functional correlates should also be taken into consideration. The quality of sleep is a well-known physiological basis of emotion regulation and stress recovery and Pittsburgh Sleep Quality Index (PSQI) has been broadly applied to mental health studies (Rezaie et al., 2023). Experimental evidence and meta-analyses have repeatedly demonstrated that lack of sleep or sleep restriction can greatly disrupt emotional states and ability to regulate emotions (Hu et al., 2023). In the population of university students, depressive symptoms were found to be significantly correlated with both the duration and quality of sleep, which implies that sleep may be an important and potentially modifiable risk factor in this group of people (Li et al., 2020). Longitudinal studies also suggest that perceived stress might mediate the connection between sleep quality and internalizing symptoms, indicating that there is an interactive relationship between sleep status and stress appraisal (Meng et al., 2025). Also, parental separation was reported as one of the contextual risks related to greater psychological distress and higher chances of developing mental health issues among university students (Nilsen et al., 2026). These extrinsic variables help to comprehend the contextual background of various psychological patterns and offer significant standards for determining the real-life relevance of latent profiles.

The current study is based on the above theoretical and methodological basis, and it uses a person-centered research paradigm and LPA to systematically analyze joint psychological features of perceived stress, anxiety, and depression in university students. Moreover, the paper explores the contribution of sleep quality and demographic variables to predicting profile membership as well as differentiating functional outcomes. In particular, this paper answers the following research questions:

*RQ1:* Do distinct latent profiles of perceived stress, anxiety, and depression exist among university students?

*RQ2:* What are the differences between various latent psychological profiles in terms of configurational patterns of perceived stress (tension and uncontrollability), anxiety, and depression?

*RQ3:* Are demographic factors and sleep quality associated with latent profile membership and differences in sleep-related functional outcomes?

## 2 Literature review

The current literature has always demonstrated that stress, anxiety, and depression are extremely co-occurring among university students and form the main expressions of mental health issues in this group. In addition to cross-cultural cross-sectional evidence, longitudinal evidence during the post-COVID period indicates that depression and anxiety can take different developmental paths as time goes by and can be affected by psychological resources related to resilience (Yang et al., 2024). Stratification of depressive symptom severity at the measurement level (e.g., PHQ-9 cutoffs) has been found to help identify high-risk individuals (Kroenke and Spitzer, 2002). In addition to severity classification, person-centered models also indicate that there may be a difference in symptom configurations in terms of psychological distress, which is structural heterogeneity across persons (Howard and Hoffman, 2017).

In terms of systems and networks, the interrelations between stress and emotional symptoms have been discovered to be characterized by cross-temporal coupling features, which means that psychological distress is not an additive process but can be a dynamically mutually enhancing process (Spătaru et al., 2024). Subsequent studies have demonstrated that depressive and anxiety symptomatology spectrum is determined by both the experiences of stress, social connectedness, and adaptive cognition, highlighting the joint influence of several psychological processes in one person (Shin and Park, 2024). Together, these results indicate that though stress, anxiety, and depression are strongly correlated with each other, they can create unique patterns of psychological configurations at the personal level.

Perceived stress is considered as one of the important psychological mechanisms connecting external environments and internal emotional reactions in mental health studies. The traditional Perceived Stress Scale (PSS) focuses on the fact that stress does not lie in the objective event itself, but rather in the subjective evaluation of life circumstances by individuals (Cohen et al., 1983). The PSS has been found to be reliable and valid in cross-cultural research, which offers a measurement basis when it comes to comparing the experiences of stress among various populations (Andreou et al., 2011).

Additional psychometric studies have revealed that perceived stress is not a one-dimensional construct, but its various dimensions can have different psychological implications. In line with this, it is valuable to identify cognitive aspects of stress in research design (Lee, 2012). The analysis of PSS subscales in older adult and community samples has demonstrated that the indicators of specific dimensions are more explanatory in predicting emotional and health outcomes (Jiang et al., 2017). Combined with cognitive appraisal theory, various dimensions of stress appraisal can be associated with emotional symptoms via various routes, which results in differentiated patterns of psychological reactions (Yeo and Ong, 2024). Thus, the incorporation of stress appraisal dimensions and emotional symptoms into a single analytical framework helps to make the concept of stress processing and its emotional effects more refined.

Even though the above research has offered a significant amount of evidence in the comprehension of psychological distress among university students, their methodological designs have been mostly variable-centered. These methods are concerned with the relationships between variables on the group-average level and implicitly suppose that people adhere to the same psychological processes (Keulen et al., 2025). Nevertheless, more recent studies have indicated that when applied to real-world situations, people who have similar levels of stress exposure or symptoms can still show significant differences in emotional reactions and functional results, which is an indicator of heterogeneity of psychological distress at the individual level (Andersson et al., 2024). In these cases, variable-based techniques tend to fail to detect joint configurational patterns of several indicators at the individual level, thus restricting further insight into the intricate organization of psychological distress (Howard and Hoffman, 2017).

Conversely, person-centered methods, including LPA, enable the modeling of several continuous indicators at once and the determination of internally homogeneous latent subgroups, which is a more suitable analytical framework to describe the heterogeneity of psychological distress structures (Wang et al., 2025). Studies based on LPA among adolescents and young adults show that depression and anxiety symptoms can be mixed in various forms instead of adhering to one pattern. Such individual variations have been applied to divide the participants into different groups according to their mental features (Aonso-Diego et al., 2024; Dai et al., 2024). Moreover, systematic correspondences between various cognitive appraisal patterns and emotional responses have also been discovered by person-centered studies of appraisal processes (Chen and Leung, 2024). However, generally speaking, most of the existing profile studies have primarily concentrated on the level of symptoms themselves and have seldom systematically incorporated cognitive aspects of perceived stress in an explicit theoretical context. Therefore, joint profile modeling of perceived stress dimensions with anxiety and depression in the context of cognitive appraisal theory is still a significant gap in the literature available today.

In addition to the psychological indicators, external functional variables are also significant in proving the real-world value of latent psychological patterns. One of the most important physiological bases of emotion regulation and stress recovery is sleep quality, and sleep issues are especially common among university students (Gardani et al., 2022). An increasing amount of empirical evidence on students in universities and health professions indicates that sleep disturbances are strongly associated with increased anxiety, and depressive symptoms (Zhang C. et al., 2024), and could partially mediate the effects of stress on mental health outcomes (Alhamed, 2023). Experimental studies and meta-analyses also show that different types of sleep loss negatively impact emotional functioning, such as lower positive affect, increased anxiety symptoms, and changed emotional reactivity, which gives strong mechanistic support to the relationship between sleep and emotions (Palmer et al., 2024).

In the case of university students, persistent sleep issues are usually addressed in conjunction with current psychological issues and decreased functioning in daily life, particularly when the students are subjected to relatively high amounts of stress (Carpi et al., 2022). Moreover, experimental research results suggest that the methods by which people regulate their emotions, including distraction or cognitive reappraisal, can affect the impact of sleep deprivation on emotional experiences, suggesting a strong relationship between sleep and processes of emotional regulation (Zhang et al., 2019). Collectively, these observations show that sleep quality is not just a significant risk factor of psychological distress but also an effective external measure of distinguishing various patterns of psychological distress. Furthermore, demographic variables, like academic year and early parental separation experience have been associated with mental health risk among university students and can offer additional information regarding the contextual backgrounds behind various latent psychological patterns. Thus, adding the quality of sleep and demographic factors to the analysis will help assess the functional importance and real-life applicability of latent psychological profiles.

## 3 Methods

### 3.1 Participants

The subjects of this research were full-time undergraduates who were registered in a comprehensive university located in the middle part of China (H University) and had various disciplinary backgrounds and all academic levels, ranging from first to fourth year. The final statistical analyses included 764 students, 468 male (61.3%) and 296 female (38.7%). The age group was 18–27 years, with an average of 20.38 years. The percentage distribution by academic year was: 231 first-year students (30.2%), 190 second-year students (24.9%), 226 third-year students (29.6%), and 117 fourth-year students (15.3%). Also, data on left-behind experience was gathered. In particular, 290 students (38.0%) said that they had experienced long-term parental absence before the age of 16 when at least one parent could not live with them during a long period, and 474 students (62.0%) said that they did not have such an experience.

### 3.2 Measures

Based on the paradigm of cognitive appraisal theory, this paper evaluated perceived stress appraisals, emotional symptoms, and sleep functioning among university students in a systematic manner. In particular, perceived stress, which is understood as the subjective cognitive evaluation of external stressors by individuals, was separated into two dimensions namely tension and uncontrollability. Internalizing emotional symptoms were described with depression and anxiety, whereas sleep quality was regarded as a significant functional outcome variable to determine the real-life applicability of various latent profiles of psychological distress.

#### 3.2.1 Perceived stress

The Perceived Stress Scale with 14 items (PSS-14) was used to measure perceived stress in this study, which was originally introduced by Cohen et al. (1983). The revised Chinese version by Yang and Huang (2003) was adopted. All the items were answered on a five-point scale with 0 (never) and 4 (always), and the higher the score, the more perceived stress there was. Instead of considering perceived stress as one construct, the PSS-14 Chinese version has two components. One of these aspects, known as tension, primarily indicates emotional responses and the degree to which everyday circumstances are perceived as stressful or dangerous. The other aspect is uncontrollability, which emphasizes how people perceive a lack of control and inadequate coping resources in the face of stressors. This two-factor model has been widely used in research on Chinese university students and has demonstrated good psychometric behavior (Huang et al., 2020). The internal consistency of the PSS-14 was satisfactory in the current sample (Cronbach’s *α* = 0.819).

#### 3.2.2 Depression

The Self-Rating Depression Scale (SDS) developed by Zung (1965) was used to measure depressive symptoms. The Chinese version revised by Zhang (1993) was employed in the study. The SDS consists of 20 items, each item being rated on a four-point response scale, with several items being scored in the reverse direction. The scores of individual items were summed up to give a total score and higher scores indicated more severe depressive symptoms. The SDS has been widely used in studies on Chinese university students and it is said to have shown acceptable reliability in previous work (Shi et al., 2024). In the present study, the scale also had good internal consistency as evidenced by a Cronbach’s alpha coefficient of 0.847.

#### 3.2.3 Anxiety

The Self-Rating Anxiety Scale (SAS), suggested by Zung (1971), according to the Chinese version, revised by Zhang (1993), was used to measure anxiety symptoms. The scale is made of 20 items that are rated on a four-point scale and some of them are reverse-scored. Item responses were added after scoring and greater total scores indicated greater levels of anxiety symptoms. SAS has been extensively used in research on Chinese adolescents and university students and it has generally been observed to have good internal consistency (Yang et al., 2024). The Cronbach’s alpha of the SAS in the current sample was high at 0.891.

#### 3.2.4 Sleep quality

The Pittsburgh Sleep Quality Index (PSQI; Buysse et al., 1989) was used to assess sleep quality. This instrument measures subjective experiences of sleep over the past month in several aspects of sleep. The scores of the different components were combined into a global sleep quality score, with higher scores indicating worse overall sleep quality. According to commonly used criteria, a global PSQI score of 6 or more was used to indicate poor sleep quality. In previous studies of Chinese university students, the PSQI has been applied and found to have acceptable reliability (Wei et al., 2025). In this study, the Cronbach’s alpha coefficient for the PSQI was 0.712.

### 3.3 Questionnaire data collection

The research was based on the cross-sectional questionnaire survey design that was conducted in a comprehensive university in central China. The population of interest included full-time undergraduate students who were in their first to fourth academic year. The online survey platform Wenjuanxing was used to collect data. The study information, electronic informed consent, and questionnaire QR code were distributed by the research team to undergraduates using academic and student affairs-related channels (e.g., course groups and class announcements) as an invitation to participate voluntarily.

Since the main purpose of the current study was to determine latent heterogeneous profiles of psychological distress in university students and to continue exploring the relationship between various profiles and external functional variables, it was more important to reflect diversity and heterogeneity in the sample than to make proportional generalizations about a larger population. In line with previous research, therefore, a non-probability convenience sampling method was used on the basis of accessibility. This is a sampling methodology that has been found to be methodologically valid in survey studies involving university students focused on LPA.

The questionnaire responses were originally obtained in a number of 956. To guarantee the quality of data, the questionnaire data were also screened and processed systematically before being analyzed as per commonly recommended practices of online survey research and data-cleaning procedures used in past studies (Lin and Yu, 2025; Wang C. L. et al., 2024; Yu et al., 2023). Specific exclusion criteria were determined and applied depending on the measurement instruments that were used and the context of the research. Such criteria were: (a) lack of information on important variables or incomplete answers to the entire battery of measures; (b) response times significantly less than a reasonable lower limit needed to finish the questionnaire; (c) obviously abnormal patterns of responses like straight-lining or logically implausible answers (e.g., sleep efficiency more than 100%); and (d) suspected duplicate submissions (e.g., submission using the same device or having abnormal submission features). All exclusions were made based on predetermined rules and finalized before any formal statistical analysis was done.

### 3.4 Data analysis

The data were analyzed with the help of SPSS version 25.0 and Mplus version 8.0. To begin with, descriptive statistical analysis was conducted to determine means, standard deviations, and internal consistency coefficients of all variables to examine the basic psychometric properties of the measurement instruments in the existing sample.

The LPA was then applied to study the clustering of various stress and emotional indicators in the sample. In particular, the results of tension, uncontrollability, anxiety, and depression were fed into the analysis as continuous measures to determine potential groupings of psychological distress among university students. The comparison of models was based on a mixture of statistical criteria including AIC, BIC, adjusted BIC, LMRT, BLRT, and entropy that are often reported in studies using LPA (Mathew and Doorenbos, 2022; Nylund et al., 2007).

Once the best latent profile model had been established, a three-step method was used to investigate the relationships between covariates and latent profile membership. In particular, multinomial logistic regression analysis was performed using the R3STEP procedure in Mplus with classification uncertainty being considered to analyze the associations of sleep quality and demographic variables (gender, academic year, and left-behind experience) with latent profile membership.

To contrast differences in sleep functioning across latent profiles, sleep quality was treated as a distal outcome variable. The Bolck-Croon-Hagenaars (BCH) method was used to estimate the mean PSQI scores for each profile and carry out between-profile comparisons (Bolck et al., 2004; Asparouhov and Muthén, 2014).

## 4 Results

### 4.1 Sample characteristics and measurement properties

The present study involved 764 undergraduate students. The sample consisted of male students who comprised 61.3 percent of the total number of students. All academic years were represented by participants, and there was a relatively larger percentage of first-year (30.2%) and third-year (29.6%) students. Regarding early family separation experiences, 38.0% of students indicated that they had experienced long absence of at least one parent prior to the age of 16, when they could not live together. It is important to note that sleep issues were common in the sample: according to the criterion of PSQI ≥6, over half of the students (54.1%) were identified as poor sleepers. Table 1 shows detailed demographic features and sleep status of the sample.

**Table 1:** Sample characteristics and sleep quality (*N* = 764).

Variable	Category	*n*	%
Sex	Male	468	61.3
	Female	296	38.7
Academic year	First year	231	30.2
	Second year	190	24.9
	Third year	226	29.6
	Fourth year	117	15.3
Left-behind experience	Yes	290	38
	No	474	62
Sleep quality (PSQI)	Poor (≥6)	413	54.1
	Good (<6)	351	45.9

PSQI ≥ 6 indicates poor sleep quality. Left-behind experience refers to prolonged parental absence before age 16.

All the psychological instruments applied in the current research were found to be acceptable to good internal consistency in the sample used (Cronbach’s *α* = 0.71–0.89). In particular, the perceived stress (PSS-14), depressive symptoms (SDS), anxiety symptoms (SAS), and sleep quality (PSQI) reliability coefficients were 0.819, 0.847, 0.891, and 0.712, respectively. These findings suggest that all the measures had acceptable measurement stability in this sample which is the minimum requirement of person-centered analyses. Considering the theoretical difference between the dimensions of perceived stress, namely tension and uncontrollability, the scores of each dimension were added as continuous indicators in the LPA. Altogether, the results of measurements can be used to identify further joint psychological patterns of stress, anxiety, and depression.

### 4.2 Identification of latent profiles

Based on the paradigm of cognitive appraisal theory, this research performed LPA based on two dimensions of perceived stress (tension and uncontrollability) and anxiety and depression scores as continuous measures. The models that identified one to four latent profiles were estimated in turn and compared in terms of model fit, quality of classification, and theoretical interpretability.

As Table 2 indicates, the increase in the number of latent profiles was accompanied by reduced AIC, BIC, and adjusted BIC values that were interpreted as an indication of enhanced model performance. Further, comparisons made using LMRT and BLRT indicated that models having two, three, or four profiles are more appropriate to fit data than models with fewer profiles (*p* < 0.05).

**Table 2:** Fit indices for latent profile models.

Model	AIC	BIC	SABIC	Entropy	LMR	BLRT	Class proportions
1	5625.599	5662.708	5637.304	–	–	–	1.00
2	4877.201	4937.502	4896.221	0.806	0	0	0.482/0.518
3	4584.141	4667.635	4610.477	0.837	<0.01	0	0.41/0.479/0.111
4	4393.919	4500.606	4427.571	0.833	0.0286	0	0.237/0.065/0.355/0.343

AIC, Akaike information criterion; BIC, Bayesian information criterion; SABIC, sample-size adjusted BIC; LMRT, Lo–Mendell–Rubin adjusted likelihood ratio test; BLRT, bootstrap likelihood ratio test.

Despite the fact that four-profile model had a slightly higher performance on information criteria than three-profile model, the three-profile solution was finally chosen as the best model following joint consideration of classification accuracy, class size, and theoretical interpretability. In particular, the entropy value obtained by using the three-profile model was relatively high (entropy = 0.837), which means that the classification accuracy is good. Also, the percentages of people in the latent profiles were fairly balanced (41.0, 47.9, and 11.1%), thus eliminating the formation of too small or unstable classes. More to the point, the profile structure that was found in the three-profile model showed obvious theoretical differentiation and could better represent different stress-emotion processing patterns as conceptualized in the context of cognitive appraisal theory. Conversely, despite marginal improvement of statistical fit indices with the four-profile model, the structure of its profile was characterized by some redundancy in the interpretation of the theory and did not offer any further substantive data.

The combination of the three-profile latent model was found to be a good compromise between the fit of the model, quality of classification, and interpretability of theory, and thus it was chosen to be used in further analysis.

### 4.3 Description of latent profile characteristics

**Figure 1:**
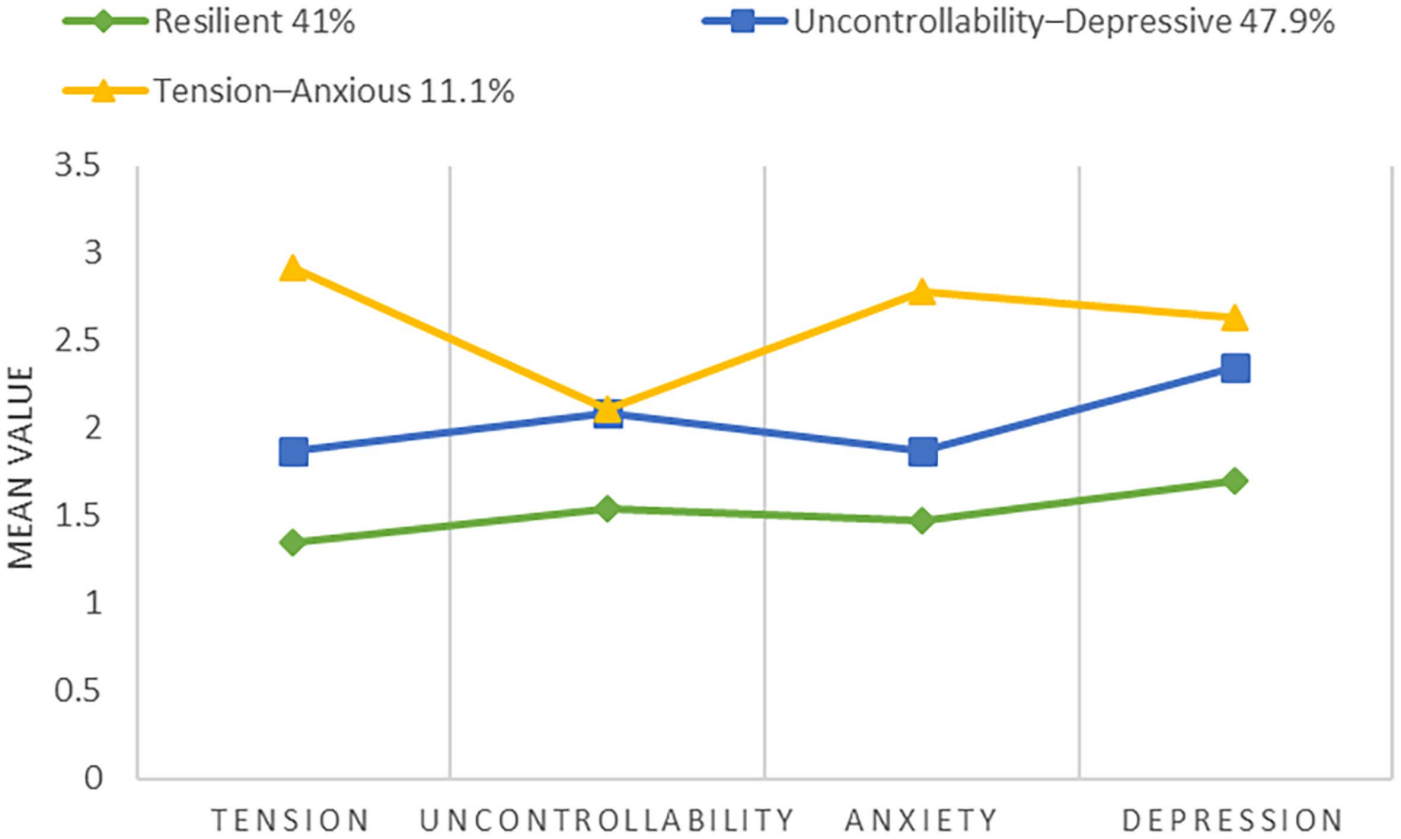
Latent profiles of perceived stress, anxiety, and depression.

The three latent profiles that were identified by the LPA had distinct and consistent differences in joint patterns of perceived stress (tension and uncontrollability), anxiety, and depression among university students (Figure 1). The comparative values of the four psychological indicators between the profiles varied not only with respect to their general magnitude but also their configuration pattern, which is an indication of unique nature of psychological processing.

#### 4.3.1 Profile 1: Resilient (41.0%, *n* = 313)

The lowest scores in all four indicators, tension (*M* = 1.351), uncontrollability (*M* = 1.546), anxiety (*M* = 1.475), and depression (*M* = 1.699) were recorded in this profile, and the general psychological distress was significantly reduced compared to the other profiles. The students of this profile were more likely to evaluate stressful situations as less threatening but had relatively stable perceptions of control and emotional states which is an indicator of greater psychological flexibility and recovery capacity. This profile was therefore termed as Resilient group.

#### 4.3.2 Profile 2: Uncontrollability-Depressive (47.9%, *n* = 366)

This profile had a moderate overall risk but showed an internal structure. In particular, uncontrollability (M = 2.094) was significantly higher than tension (M = 1.868), and depression levels (M = 2.352) were more than anxiety levels (M = 2.053). This trend indicates that students in this profile mainly encountered stress in the cognitive appraisals of inadequate coping resources and control deficiency, with emotional reactions tending to be helpless and low mood instead of high-arousal anxiety. Based on this, this group was termed as Uncontrollability-Depressive group.

#### 4.3.3 Profile 3: Tension-Anxious (11.1%, *n* = 85)

This profile was the most intense in all four psychological indicators and was characterized by especially high tension (*M* = 2.915) and anxiety (*M* = 2.781), which were significantly higher than uncontrollability (*M* = 2.107) and depression (*M* = 2.637). This trend shows that students of this profile tend to evaluate stressful events as highly threatening and have prolonged high-arousal emotional reactions. In line with a high-risk psychological pattern, which is dominated by threat appraisal and anxiety responses, this profile has been referred to as the *Tension-Anxious* group.

In general, these three latent profiles showed not only a graded increase in the severity of psychological distress between low and high but also qualitative differences in the configurational patterns of stress appraisals and emotional responses. These results combined give person-centered data on the heterogeneity of psychological distress among university students.

### 4.4 Predictors of latent profile membership

To initially explain the distribution of demographic variables among latent profiles, chi-square tests were performed to examine differences in gender, academic year, and left-behind experience between the three latent profiles. The results are presented in Table 3.

**Table 3:** Distribution of sample characteristics across latent profiles.

Variable	Category	C1 Resilient (*n* = 313)	C2 Uncontrollability-Depressive (*n* = 366)	C3 Tension-Anxious (*n* = 85)	*χ* ^2^	*p*
Sex	Male	191 (61.0)	225 (61.5)	52 (61.2)	0.015	0.993
	Female	122 (39.0)	141 (38.5)	33 (38.8)		
Academic year	First year	110 (35.1)	107 (29.2)	14 (16.5)	16.876	<0.05
	Second year	78 (24.9)	84 (23.0)	28 (32.9)		
	Third year	75 (24.0)	120 (32.8)	31 (36.5)		
	Fourth year	50 (16.0)	55 (15.0)	12 (14.1)		
Left-behind experience	Yes	90 (28.8)	155 (42.3)	45 (52.9)	22.36	<0.001
	No	223 (71.2)	211 (57.7)	40 (47.1)		

Values are *n* (%).

The findings showed that there were substantial variations in the academic year distribution across the latent profiles (*χ*^2^ = 16.876, *p* < 0.05). In general, the percentage of students with the Resilient profile was found to be decreasing as the academic year progressed and third-year students were comparatively overrepresented in the *Uncontrollability-Depressive* and *Tension-Anxious* profiles.

Also, latent profile membership was strongly linked to the left-behind experience (*χ*^2^ = 22.36, *p* < 0.001). In comparison with students who did not report having a left-behind experience, students who reported such an experience were more likely to be placed in higher-risk profiles, and the most significant overrepresentation was found in the *Tension-Anxious* profile. Conversely, there were no statistically significant differences between the distribution of gender across the three latent profiles (*χ*^2^ = 0.015, *p* = 0.993).

It is important to note that the above results were based on bivariate descriptive comparisons and did not consider classification uncertainty in latent profile assignment or the interrelationships among covariates. The main purpose of these analyses was to describe the demographic distribution of the latent profiles and to provide contextual background for subsequent multivariable predictive analyses.

According to this premise, the current research also used the three-step method (R3STEP) in Mplus to test the independent predictive impacts of sleep quality and demographic variables on latent profile membership taking into consideration the uncertainty of classification. The findings of the multinomial logistic regression analyses are presented as odds ratios (ORs), 95% confidence intervals, and *p* values with the Resilient profile as the reference group, as indicated in Table 4.

**Table 4:** Multinomial logistic regression predicting latent profile membership.

Predictor	Comparison	OR	95% CI	*p*
Sex	C2 vs. C1	0.986	[0.665, 1.462]	0.944
	C3 vs. C1	0.704	[0.347, 1.429]	0.244
Academic year	C2 vs. C1	1.109	[0.929, 1.324]	0.278
	C3 vs. C1	1.197	[0.875, 1.637]	0.304
Left-behind experience	C2 vs. C1	1.495	[0.992, 2.255]	0.114
	C3 vs. C1	1.989	[0.980, 4.035]	0.168
PSQI	C2 vs. C1	1.42	[1.311, 1.537]	<0.001
	C3 vs. C1	2.202	[1.872, 2.588]	<0.001

C1 = resilient; C2 = uncontrollability-depressive; C3 = tension-anxious. OR, odds ratio; CI, confidence interval.

The findings revealed that the sleep quality was the most consistent predictor of latent profile membership and had the biggest effect size. In particular, every one-point increment in PSQI score raised the probability of being categorized into the Uncontrollability Depressive profile significantly [OR = 1.420, 95% CI (1.311, 1.537), *p* < 0.001], but the rise in the probability of being placed in the Tension Anxious profile was even greater [OR = 2.202, 95% CI (1.872, 2.588), *p* < 0.001]. These results show a high and strong relationship between lower quality of sleep and belonging to more risky psychological distress profiles.

However, once sleep quality and other covariates were controlled, left-behind experience, academic year, and gender did not have significant predictive effects on latent profile membership (all ps > 0.05). This finding indicates that despite the association between left-behind experience and academic year with profile membership in bivariate analyses, these factors might be explained by more proximal risk factors like sleep quality in multivariable analysis.

### 4.5 Sleep quality differences across latent profiles

Sleep quality was also considered as a functional distal outcome variable, and the BCH method was used to examine differences in PSQI scores between the latent profiles. The results are presented in Table 5.

**Table 5:** Sleep quality across latent profiles.

Profile	PSQI mean	SE
C1	4.279	0.146
C2	6.637	0.162
C3	10.42	0.448

C1 = resilient; C2 = uncontrollability-depressive; C3 = tension-anxious. Means (SE) were estimated using the BCH procedure. Overall Wald test: *χ*^2^(2) = 243.176, *p* < 0.001.

The findings revealed that there were notable variations in the quality of sleep among the latent profiles. According to the overall Wald *χ*^2^ test estimated by BCH, PSQI scores varied significantly between the three latent profiles [*χ*^2^(2) = 243.176, *p* < 0.001]. In particular, the *Resilient* profile had the lowest mean PSQI score which means relatively improved general sleep quality. The *Uncontrollability-Depressive* profile exhibited a moderately increased level of PSQI, and the *Tension-Anxious* profile showed the highest mean PSQI score, indicating the most serious deterioration in the functioning of sleep. It was also found that all the differences among the three profiles were significant (all ps < 0.001) and the biggest difference was recorded between the *Tension-Anxious* and *Resilient* profiles.

These results were used to extend the differences in the risk of poor sleep quality between latent profiles with a public health and applied perspective. The findings revealed that, at a commonly used clinical cut-off point of PSQI 6 or more, there was an obvious graded distribution of the risk of poor sleep quality across the three latent profiles. The majority of people in the Resilient profile scored normally on the PSQI. Conversely, the percentage of poor sleep quality was significantly greater among the *Uncontrollability-Depressive* profile whereas the *Tension-Anxious* profile had the greatest amount of sleep-related risk with average PSQI scores much higher than the clinical threshold. These results also show that the psychological distress profiles that are considered as high-risk are not only marked by high emotional and stress indicators but also by a considerably more severe disruption in the functioning of sleep.

## 5 Discussion

### 5.1 Principal findings and profile interpretation

According to a person-centered analytical paradigm, the current study has revealed three different latent profiles of psychological distress among university students: *Resilient*, *Uncontrollability-Depressive*, and *Tension-Anxious*. This observation implies that there is no uniform distribution of psychological distress in university students along a single continuum of severity but instead shows high heterogeneity in the configurational patterns of stress appraisals and emotional reactions. This outcome goes beyond previous research methods that have largely described student mental health as overall means or variable-level relationships, and is aligned with existing systematic reviews that point to the general prevalence of psychological stress among students and significant within-group variation (Fang et al., 2022). Also, the findings presented are very close to recent studies on latent profiles carried out by undergraduate nursing students, which further confirms the suitability of person-centered approaches in the context of university mental health research (Wang et al., 2025).

It is important to note that the three profiles did not only vary in terms of general levels of psychological distress, which had a graded distribution between low and high, but also qualitative differences in the combinations of cognitive elements of perceived stress and emotional symptoms. The *Resilient* profile was defined by the presence of low tension, uncontrollability, anxiety, and depression, indicating that it has a relatively balanced pattern of stress appraisal and stable functioning of emotions. This trend is aligned with the past results that have pointed out self-efficacy, resilience, and positive psychological resources as major buffers against stress (Berdida et al., 2023; Wang Q. et al., 2024). Conversely, the *Uncontrollability-Depressive* profile was characterized by increased uncontrollability and depressive symptoms, which implies that the experiences of stress in this group were mainly caused by cognitive evaluations of inadequate coping resources and lack of control. Such trends have been found among Chinese nursing students where these appraisals were closely linked to maladaptive coping styles and emotional burnout (Ma et al., 2022), and they are consistent with findings regarding the mediating effect of perceived stress on the formation of depressive symptoms (Arrivillaga et al., 2022). In comparison, the *Tension-Anxious* profile showed high levels of tension and anxiety with high-arousal ratings of situational threat being sustained. This anxiety-dominant pattern aligns with previous studies demonstrating that increased emotional arousal is an essential aspect of the underlying structure of anxiety-related symptoms and behavioral risk dispositions (Yang et al., 2025).

The profile structure that was identified shows high conceptual consistency with the cognitive appraisal theory of Lazarus. Within this framework, it is not situational characteristics that dictate stress responses, but rather a combination between primary threat appraisals and secondary coping resources appraisals. In the current study, profiles characterized by increased tension were more prone to be accompanied by anxiety reactions, whereas those focused on uncontrollability were more closely related to depressive experiences, indicating that different appraisal pathways can lead to various emotional processing effects. This explanation is in line with previous studies showing that cognitive appraisals affect depressive symptoms via perceived stress and self-efficacy (Liu et al., 2023), as well as multidimensional models placing cognitive appraisal processes in larger contexts connecting technostress with learning outcomes (Sharma and Gupta, 2023). Moreover, research on cognitive intervention based on perceived stress and depression has demonstrated that changing the way people cognitively process stressful situations can simultaneously enhance the experience of stress and emotional conditions, which provides further support to the modifiability of appraisal pathways (Zhu et al., 2022). Combined, the current paper provides new empirical evidence of the person-centered applicability of cognitive appraisal theory to university students and also proves that when similar stress circumstances are involved, people can develop different types of psychological distress due to differentiated psychological processing mechanisms.

### 5.2 Differential associations with sleep and demographic risk

The current research was based on the recognition of latent psychological profiles and further explored the relations between sleep quality and demographic variables with profile membership and functional outcomes. The findings revealed that sleep quality was at the center of predicting both profile membership and differentiating the profiles. Multivariable analyses indicated that worse sleep quality was significantly related to a greater probability of being part of the two high-risk profiles, especially the *Tension-Anxious* profile, and this relationship was strong even after adjusting for gender, academic year, and left-behind experience. This result is in line with previous research which has applied latent profile methods to study heterogeneity in sleep, showing that sleep quality itself can be differentiated into subtypes with clinical and functional relevance and it is highly correlated with psychological symptoms like anxiety and depression (Pan et al., 2024; Xie et al., 2024). In addition, sleep profiles have also been found to be systematically associated with psychosocial risk factors and emotional distress levels in other health-related populations, which again supports the key position of sleep in the organization of psychological risk (Li et al., 2023).

The BCH analyses results also showed that there were clear and graded differences in sleep functioning among the latent profiles. The *Resilient* profile was relatively good in terms of sleep quality, the *Uncontrollability-Depressive* profile had a moderate level of sleep-related risk, and the *Tension-Anxious* profile demonstrated the worst impairments in the functioning of sleep. These observations confirm the real-life applicability of the latent profiles in terms of functional outcome because it can be seen that the profiles are not just statistical differences but represent some significant variations in everyday activities which people can perceive. Also, stable hierarchical patterns of functional impairment across various psychological or sleep profiles have been reported in previous studies in clinical and occupational samples, which is another argument in favor of a profile-based approach to mental health disparities (Pan et al., 2024; Xie et al., 2024).

Mechanically, the observed correlations between various profiles and sleep have a distinct theoretical plausibility. People with high levels of tension and anxiety tend to be in a constant state of hyperarousal and threat surveillance, and it has been established that hyperarousal is one of the key processes that cause insomnia and sleep continuity issues (Dressle and Riemann, 2023). Similarly, people who are dominated by uncontrollability appraisals and depressive experiences are more susceptible to rumination, helplessness, and problems with emotion regulation, which have been demonstrated to interfere with sleep maintenance and worsen sleep instability (Mombelli et al., 2025). According to experience-sampling research, not only does the decline in sleep quality affect the emotional experiences per se but also reduces the ability of individuals to control positive emotions, thus enhancing negative emotional reactions (Parsons et al., 2022). Collectively, these results indicate that sleep impairment and emotional distress can create a mutually reinforcing negative cycle that is integrated into the dynamic mechanisms of stress appraisal and emotional responding.

On the other hand, the demographic variables had no significant independent predictive effects in the multivariable models. Though academic year and left-behind experience were linked to profile membership at the bivariate level, they ceased to have a significant impact when sleep quality was controlled, indicating that these background factors could have an indirect effect on patterns of psychological distress via sleep and other more proximal psychological processes. Previous studies indicate that the mental health consequences of early adverse experiences and family-related risk backgrounds can be mediated indirectly by more immediate mechanisms like sleep disruptions and challenges with emotion regulation (Jiang et al., 2021). Furthermore, intervention research focusing on people suffering from severe mental disorders has shown that better sleep and circadian rhythms may partially counteract the negative impacts of pre-existing risk backgrounds on psychological outcomes, which is why sleep should be considered as one of the modifiable intervention targets (Armstrong et al., 2022). Altogether, the results of the current paper reinforce the fact that sleep quality plays the central role in the organization of psychological distress among university students.

### 5.3 Implications, limitations, and future directions

The results of the current study have theoretical and practical implications regarding the structural features of psychological distress among university students, as well as possible intervention points. On the theoretical level, based on cognitive appraisal theory and using a person-centered approach, the present study explained how various cognitive elements of perceived stress (tension and uncontrollability) interact with anxiety and depression to create unique configurational patterns in the same population. In contrast to traditional variable-based analyses that examine overall associations or average effects, the findings presented here show that even when individuals are exposed to similar stressful situations, they can develop psychological response patterns characterized by high-arousal anxiety or depressive experiences through different appraisal pathways. These results provide evidence at the configurational level for cognitive appraisal theory, indicating that stress appraisals and emotional symptoms do not accumulate linearly at the individual level but rather coexist in heterogeneous structural forms.

At the applied level, the findings indicate that university mental health services can potentially gain by changing their mean-level severity-oriented risk assessments to more differentiated profile-based identification strategies. The latent psychological profiles found in the current research demonstrated systematic differences in stress appraisal patterns, emotional reactions, and intensity of sleep-related functional impairment, which means that a single-size-fits-all intervention strategy might not be enough to meet the needs of students. It is interesting to note that sleep quality was the most relevant predictor of profile membership and outcome differentiation. Despite the inability to make causal inferences based on the results obtained in the current study, the findings indicate that sleep, due to its low stigma, ease of measurement, and high modifiability, can be used as an effective starting point for psychological risk screening and early intervention in universities.

There are a number of shortcomings in the current study that should be interpreted with care. To begin with, it is not possible to make conclusions about the temporal order and causal relations between latent profiles and sleep quality due to the cross-sectional design. Longitudinal or experimental studies would be required to investigate the possibility of bidirectional or dynamic relationships among these variables in future research. Second, the sample was based on one university. Even though the size of the sample was sufficient and students were recruited at various academic levels, the results must be considered in terms of generalizability to multi-site or cross-cultural samples. Moreover, all the variables were measured by means of self-report questionnaires, which can be prone to common method bias. Objective sleep measures, behavioral information, or interview-based measurements may be added to future studies to increase the validity of multimethod measurement.

Considering these limitations, a few directions of future research are proposed. The latent psychological profiles that were determined in this study can be used as the basis of longitudinal studies. In future research, latent transition analysis can be used to examine systematically the stability and transitions between psychological profiles over time, and also to determine important risk and protective factors related to profile change. In addition, it would be desirable to include more functional and behavioral indicators (academic adjustment, help-seeking behavior, emotion regulation strategies or physiological response measures) to better understand the real-world implications of various psychological profiles and learning processes. Future studies could provide more empirically-based advice on accurate assessment and stratified intervention in university mental health settings by combining cognitive appraisal processes, profile structures and functional outcomes.

## 6 Conclusion

The current research was conducted systematically according to the person-centered analytical paradigm and explored the joint psychological traits of perceived stress, anxiety, and depression in university students and found well-defined latent psychological profiles. The results show that the psychological distress among university students is not a single continuum of severity but has significant heterogeneity in the patterns of configuration of stress appraisal components and emotional symptoms. These various latent profiles were not only different in terms of overall distress levels, but also in consistent and psychologically relevant structural differences between the way cognitive appraisal dimensions (tension and uncontrollability) interacted with anxiety and depression, which demonstrated various forms of stress processing and responding to emotions at the individual level.

Additional investigations showed that sleep quality was especially relevant in distinguishing both psychological profiles and their functional expressions. The membership to higher-risk psychological profiles and more severe impairments of the functioning related to sleep were always linked with poorer sleep quality, whereas demographic background variables did not have independent predictive effects when controlling by sleep quality. These results indicate that the risk profile of psychological distress among university students can be more significantly influenced by proximal psychological and physiological processes rather than relatively stable demographic characteristics.

In general, the study contributes to a person-focused knowledge of heterogeneity in psychological distress among university students by combining cognitive appraisal processes, configurational patterns of emotional symptoms, and functional outcomes. The results not only offer structural-level empirical evidence of the use of cognitive appraisal theory in university populations but also provide practically useful data that can be used to inform stratified risk assessment and focused intervention in university mental health services.

## Data Availability

The original contributions presented in the study are included in the article/supplementary material, further inquiries can be directed to the corresponding author.

## References

[ref1] Alhamed A. A. (2023). The link among academic stress, sleep disturbances, depressive symptoms, academic performance, and the moderating role of resourcefulness in health professions students during COVID-19 pandemic. J. Prof. Nurs. 46, 83–91. doi: 10.1016/j.profnurs.2023.02.010, 37188428 PMC10020862

[ref2] Alwhaibi M. Alotaibi A. Alsaadi B. (2023). Perceived stress among healthcare students and its association with anxiety and depression: a cross-sectional study in Saudi Arabia. Healthcare (Basel) 11:1625. doi: 10.3390/healthcare11111625, 37297765 PMC10252950

[ref3] Andersson J. Kankaanpää R. Peltonen K. Münger A. C. Korhonen L. (2024). Examining heterogeneity: a systematic review of quantitative person-centered studies on adversity, mental health, and resilience in children and young adults with refugee backgrounds. Compr. Psychiatry 135:152522. doi: 10.1016/j.comppsych.2024.152522, 39142243

[ref4] Andreou E. Alexopoulos E. C. Lionis C. Varvogli L. Gnardellis C. Chrousos G. P. . (2011). Perceived stress scale: reliability and validity study in Greece. Int. J. Environ. Res. Public Health 8, 3287–3298. doi: 10.3390/ijerph8083287, 21909307 PMC3166743

[ref5] Aonso-Diego G. González-Roz A. Weidberg S. Secades-Villa R. (2024). Depression, anxiety, and stress in young adult gamers and their relationship with addictive behaviors: a latent profile analysis. J. Affect. Disord. 366, 254–261. doi: 10.1016/j.jad.2024.08.203, 39218313

[ref6] Armstrong C. C. Dong L. Harvey A. G. (2022). Mediators and moderators of outcome from the Transdiagnostic Sleep and Circadian Intervention for adults with severe mental illness in a community setting. Behav. Res. Ther. 151:104053. doi: 10.1016/j.brat.2022.104053, 35152036

[ref7] Arrivillaga C. Rey L. Extremera N. (2022). A mediated path from emotional intelligence to problematic social media use in adolescents: the serial mediation of perceived stress and depressive symptoms. Addict. Behav. 124:107095. doi: 10.1016/j.addbeh.2021.107095, 34479068

[ref8] Asparouhov T. Muthén B. O. (2014). Auxiliary variables in mixture modeling: three-step approaches using Mplus. Struct. Equ. Model. 21, 329–341. doi: 10.1080/10705511.2014.915181

[ref9] Berdida D. J. E. Lopez V. Grande R. A. N. (2023). Nursing students' perceived stress, social support, self-efficacy, resilience, mindfulness and psychological well-being: a structural equation model. Int. J. Ment. Health Nurs. 32, 1390–1404. doi: 10.1111/inm.13179, 37249199

[ref10] Bolck A. Croon M. Hagenaars J. (2004). Estimating latent structure models with categorical variables: one-step versus three-step estimators. Polit. Anal. 12, 3–27. doi: 10.1093/pan/mph001

[ref11] Bonanno G. A. (2004). Loss, trauma, and human resilience: have we underestimated the human capacity to thrive after extremely aversive events? Am. Psychol. 59, 20–28. doi: 10.1037/0003-066X.59.1.20, 14736317

[ref12] Buysse D. J. Reynolds C. F. Monk T. H. Berman S. R. Kupfer D. J. (1989). The Pittsburgh sleep quality index: a new instrument for psychiatric practice and research. Psychiatry Res. 28, 193–213. doi: 10.1016/0165-1781(89)90047-4, 2748771

[ref13] Carpi M. Cianfarani C. Vestri A. (2022). Sleep quality and its associations with physical and mental health-related quality of life among university students: a cross-sectional study. Int. J. Environ. Res. Public Health 19:2874. doi: 10.3390/ijerph19052874, 35270566 PMC8910365

[ref14] Chen X. Leung F. K. (2024). Secondary school students' appraisal profiles and their relations with academic emotions in mathematics. Learn. Individ. Differ. 115:102545. doi: 10.1016/j.lindif.2024.102545

[ref15] Cohen S. Kamarck T. Mermelstein R. (1983). A global measure of perceived stress. J. Health Soc. Behav. 24, 385–396. doi: 10.2307/2136404, 6668417

[ref16] Dai Y. Zheng Y. Hu K. Chen J. Lu S. Li Q. . (2024). Heterogeneity in the co-occurrence of depression and anxiety among adolescents: results of latent profile analysis. J. Affect. Disord. 357, 77–84. doi: 10.1016/j.jad.2024.04.065, 38670464

[ref17] Dressle R. J. Riemann D. (2023). Hyperarousal in insomnia disorder: current evidence and potential mechanisms. J. Sleep Res. 32:e13928. doi: 10.1111/jsr.13928, 37183177

[ref18] Estrella-Proaño A. Rivadeneira M. F. Alvarado J. Murtagh M. Guijarro S. Alomoto L. . (2024). Anxiety and depression in first-year university students: the role of family and social support. Front. Psychol. 15:1462948. doi: 10.3389/fpsyg.2024.1462948, 39649784 PMC11621471

[ref19] Fang Y. Ji B. Liu Y. Zhang J. Liu Q. Ge Y. (2022). The prevalence of psychological stress in student populations during the COVID-19 epidemic: a systematic review and meta-analysis. Sci. Rep. 12:12118. doi: 10.1038/s41598-022-16328-7, 35840641 PMC9284967

[ref20] Fauzi M. F. Anuar T. S. Teh L. K. Lim W. F. James R. J. Ahmad R. . (2021). Stress, anxiety and depression among a cohort of health sciences undergraduate students: the prevalence and risk factors. Int. J. Environ. Res. Public Health 18:3269. doi: 10.3390/ijerph18063269, 33809939 PMC8004268

[ref21] Feng G. Xu X. Lei J. (2023). Tracking perceived stress, anxiety, and depression in daily life: a double-downward spiral process. Front. Psychol. 14:1114332. doi: 10.3389/fpsyg.2023.1114332, 37143594 PMC10151810

[ref22] Fentahun S. Rtbey G. Nakie G. Andualem F. Tinsae T. Kibralew G. . (2025). Burden of perceived stress among university students in Africa: a systematic review and meta-analysis. BMC Public Health 25:2248. doi: 10.1186/s12889-025-23533-2, 40604771 PMC12220081

[ref23] Fried E. I. (2022). Studying mental health problems as systems, not syndromes. Curr. Dir. Psychol. Sci. 31, 500–508. doi: 10.1177/09637214221114089

[ref24] Gardani M. Bradford D. R. Russell K. Allan S. Beattie L. Ellis J. G. . (2022). A systematic review and meta-analysis of poor sleep, insomnia symptoms and stress in undergraduate students. Sleep Med. Rev. 61:101565. doi: 10.1016/j.smrv.2021.101565, 34922108

[ref25] Garland E. Gaylord S. Park J. (2009). The role of mindfulness in positive reappraisal. Explore (NY) 5, 37–44. doi: 10.1016/j.explore.2008.10.001, 19114262 PMC2719560

[ref26] Howard M. C. Hoffman M. E. (2017). Variable-centered, person-centered, and person-specific approaches: where theory meets the method. Organ. Res. Methods 21, 846–876. doi: 10.1177/1094428117744021

[ref27] Hu Y. Liu J. Zhao Z. Bi C. Cao H. Liu H. . (2023). Association between sleep quality and psychological symptoms: a cross-sectional survey of Chinese university students performed during the COVID-19 pandemic. Front. Psychol. 14:1131176. doi: 10.3389/fpsyg.2023.1131176, 37260956 PMC10227667

[ref28] Huang F. Wang H. Wang Z. Zhang J. Du W. Su C. . (2020). Psychometric properties of the perceived stress scale in a community sample of Chinese. BMC Psychiatry 20:130. doi: 10.1186/s12888-020-02520-4, 32197589 PMC7082906

[ref29] Ike O. O. Kwarteng M. A. Ogbonna G. Dos Santos I. B. Ogiemudia O. M. Anyasodor A. E. . (2025). Cross-national variations in mental health: a cross-sectional study on depression, anxiety, and stress among university staff and students in Sub-Saharan African. PLoS One 20:e0322163. doi: 10.1371/journal.pone.0322163, 40460138 PMC12133007

[ref30] Jiang J. Seng E. Zimmerman M. Sliwinski M. Kim M. Lipton R. (2017). Evaluation of the reliability, validity, and predictive validity of the subscales of the perceived stress scale in older adults. J. Alzheimer's Dis 59, 987–996. doi: 10.3233/JAD-170289, 28671128 PMC5777162

[ref31] Jiang L. Shi X. Wang Z. Wang S. Li Z. Wang A. (2021). Sleep problems and emotional dysregulation mediate the relationship between childhood emotional abuse and suicidal behaviors: a three-wave longitudinal study. J. Affect. Disord. 295, 981–988. doi: 10.1016/j.jad.2021.09.003, 34706472

[ref32] Kennedy S. M. Tonarely N. A. Halliday E. Ehrenreich-May J. (2022). A person-centered approach to understanding heterogeneity of youth receiving transdiagnostic treatment for emotional disorders. J. Consult. Clin. Psychol. 90, 234–245. doi: 10.1037/ccp0000710, 35175069

[ref33] Keulen J. Deković M. Vervoort L. Bodden D. (2025). Exploring transdiagnostic factors of internalizing and externalizing symptoms in transitional-age youth: using a variable-centered and person-centered approach. Curr. Psychol. 44, 16866–16882. doi: 10.1007/s12144-025-08328-3, 41199989 PMC12586208

[ref34] Kivity Y. Huppert J. D. (2016). Does cognitive reappraisal reduce anxiety? A daily diary study of a micro-intervention with individuals with high social anxiety. J. Consult. Clin. Psychol. 84, 269–283. doi: 10.1037/ccp0000075, 26795939

[ref35] Kroenke K. Spitzer R. L. (2002). The PHQ-9: a new depression diagnostic and severity measure. Psychiatr. Ann. 32, 509–515. doi: 10.3928/0048-5713-20020901-06

[ref36] Lazarus R. S. Folkman S. (1984). Stress, Appraisal, and Coping. New York: Springer.

[ref37] Lee E.-H. (2012). Review of the psychometric evidence of the perceived stress scale. Asian Nurs. Res. 6, 121–127. doi: 10.1016/j.anr.2012.08.004, 25031113

[ref38] Li S. Wang X. Wang M. Jiang Y. Mai Q. Wu J. . (2023). Association between stigma and sleep quality in patients with breast cancer: a latent profile and mediation analysis. Eur. J. Oncol. Nurs. 67:102453. doi: 10.1016/j.ejon.2023.102453, 37951070

[ref39] Li W. Yin J. Cai X. Cheng X. Wang Y. (2020). Association between sleep duration and quality and depressive symptoms among university students: a cross-sectional study. PLoS One 15:e0238811. doi: 10.1371/journal.pone.0238811, 32915844 PMC7485879

[ref40] Lin C. H. Siao S. F. Lin Y. J. Hsin P. H. Shelley M. Lee Y. H. (2023). Cognitive appraisals and coping strategies of registered nurses in the emergency department combating COVID-19: a scoping review. J. Nurs. Scholarsh. 55, 79–96. doi: 10.1111/jnu.12815, 36138561 PMC9538970

[ref41] Lin Y. Yu Z. (2025). Elucidating university students’ intentions to seek automated writing feedback from Grammarly: toward perceptual and systemic predictors. Humanit. Soc. Sci. Commun. 12:7. doi: 10.1057/s41599-024-03861-1

[ref42] Liu Y. Wen S. S. Chen Y. Zheng J. W. Xiao H. M. (2023). Cognitive appraisal and depression in cancer patients undergoing chemotherapy: mediation by perceived stress and self-efficacy. Support. Care Cancer 31:614. doi: 10.1007/s00520-023-08075-w, 37801183

[ref43] Lubke G. Muthén B. O. (2007). Performance of factor mixture models as a function of model size, covariate effects, and class-specific parameters. Struct. Equ. Model. Multidiscip. J. 14, 26–47. doi: 10.1080/10705510709336735

[ref44] Luo Z. Shen Y. Yuan J. Zhao Y. Liu Z. Shangguan F. (2021). Perceived stress, resilience, and anxiety among pregnant Chinese women during the COVID-19 pandemic: latent profile analysis and mediation analysis. Front. Psychol. 12:696132. doi: 10.3389/fpsyg.2021.696132, 34367022 PMC8339262

[ref45] Ma H. Zou J. M. Zhong Y. Li J. He J. Q. (2022). Perceived stress, coping style and burnout of Chinese nursing students in late-stage clinical practice: a cross-sectional study. Nurse Educ. Pract. 62:103385. doi: 10.1016/j.nepr.2022.103385, 35780686

[ref46] Mathew A. Doorenbos A. Z. (2022). Latent profile analysis–an emerging advanced statistical approach to subgroup identification. Indian J. Contin. Nurs. Educ. 23, 127–133. doi: 10.4103/ijcn.ijcn_24_22

[ref47] Meng R. Xu J. Luo Y. Mastrotheodoros S. Jiang C. Garofalo C. . (2025). Perceived stress mediates the longitudinal effect of sleep quality on internalizing symptoms. J. Affect. Disord. 373, 51–59. doi: 10.1016/j.jad.2024.12.046, 39675679

[ref48] Miao H. Wei Z. Li Q. Zhang Y. Liu X. Guo C. (2024). Psychological *Suzhi* and depression and anxiety among Chinese adolescents: the mediating role of negative cognitive processing bias and perceived stress. Curr. Psychol. 43, 18207–18217. doi: 10.1007/s12144-023-05582-1

[ref49] Mombelli S. Fasiello E. Di Perri M. C. Casoni F. Zucconi M. Ferini-Strambi L. . (2025). The interplay between emotion dysregulation and repetitive thoughts in insomnia disorder: the impact of worry, rumination and REM sleep instability. J. Sleep Res. 14:e70267. doi: 10.1111/jsr.70267, 41392615 PMC13357897

[ref50] Nilsen S. A. Hysing M. Sivertsen B. (2026). Parental separation and mental health problems among university students. J. Affect. Disord. 397:121010. doi: 10.1016/j.jad.2025.121010, 41422959

[ref51] Nylund K. L. Asparouhov T. Muthén B. O. (2007). Deciding on the number of classes in latent class analysis and growth mixture modeling: a Monte Carlo simulation study. Struct. Equ. Model. Multidiscip. J. 14, 535–569. doi: 10.1080/10705510701575396

[ref52] Paiva U. Cortese S. Flor M. Moncada-Parra A. Lecumberri A. Eudave L. . (2025). Prevalence of mental disorder symptoms among university students: an umbrella review. Neurosci. Biobehav. Rev. 175:106244. doi: 10.1016/j.neubiorev.2025.106244, 40480638

[ref53] Palmer C. A. Bower J. L. Cho K. W. Clementi M. A. Lau S. Oosterhoff B. . (2024). Sleep loss and emotion: a systematic review and meta-analysis of over 50 years of experimental research. Psychol. Bull. 150, 440–463. doi: 10.1037/bul0000410, 38127505

[ref54] Pan X. Wang J. Zhang K. Yang C. Tang M. Feng Z. . (2024). Characterising potential subtypes and influencing factors of sleep quality in psychiatric nurses by latent profile analysis. J. Nurs. Manag. 2024:3842592. doi: 10.1155/2024/3842592 40224797 PMC11919122

[ref55] Parsons C. E. Schofield B. Batziou S. E. Ward C. Young K. S. (2022). Sleep quality is associated with emotion experience and adaptive regulation of positive emotion: an experience sampling study. J. Sleep Res. 31:e13533. doi: 10.1111/jsr.13533, 35896512

[ref56] Pedersen H. S. Christensen K. S. Prior A. Christensen K. B. (2024). The dimensionality of the perceived stress scale: the presence of opposing items is a source of measurement error. J. Affect. Disord. 344, 485–494. doi: 10.1016/j.jad.2023.10.109, 37852582

[ref57] Rezaie L. Norouzi E. Bratty A. J. Khazaie H. (2023). Better sleep quality and higher physical activity levels predict lower emotion dysregulation among persons with major depression disorder. BMC Psychol. 11:171. doi: 10.1186/s40359-023-01213-3, 37226277 PMC10207795

[ref58] Saqr M. Vogelsmeier L. V. Lopez-Pernas S. (2024). Capturing where the learning process takes place: a person-specific and person-centered primer. Learn. Individ. Differ. 113:102492. doi: 10.1016/j.lindif.2024.102492

[ref59] Sharma S. Gupta B. (2023). Investigating the role of technostress, cognitive appraisal and coping strategies on students' learning performance in higher education: a multidimensional transactional theory of stress approach. Inf. Technol. People 36, 626–660. doi: 10.1108/ITP-06-2021-0505

[ref60] Shi Z. Chen H. Zhang Y. Guan J. Xia B. Jin Z. (2024). The effect of parental helicopter parenting on subjective well-being of college students: the chain mediating effect of self-control and depression. Stud. Psychol. Behav. 22, 116–122. doi: 10.12139/j.1672-0628.2024.01.016

[ref61] Shin H. Park C. (2024). Mastery is central: an examination of complex interrelationships between physical health, stress and adaptive cognition, and social connection with depression and anxiety symptoms. Front. Psych. 15:1401142. doi: 10.3389/fpsyt.2024.1401142, 38751422 PMC11094708

[ref62] Slimmen S. Timmermans O. Mikolajczak-Degrauwe K. Oenema A. (2022). How stress-related factors affect mental wellbeing of university students a cross-sectional study to explore the associations between stressors, perceived stress, and mental wellbeing. PLoS One 17:e0275925. doi: 10.1371/journal.pone.0275925, 36342914 PMC9639818

[ref63] Spătaru B. Podină I. R. Tulbure B. T. Maricuțoiu L. P. (2024). A longitudinal examination of appraisal, coping, stress, and mental health in students: a cross-lagged panel network analysis. Stress. Health 40:e3450. doi: 10.1002/smi.3450, 39037706

[ref64] Stokoe M. Nordstokke D. Wilcox G. (2024). First year students' perceptions of the transition to university: the role of informational, instrumental, and emotional support. Int. J. Res. Educ. Sci. 10, 377–393. doi: 10.46328/ijres.3392

[ref65] Wang Y. Deng Q. Fu Y. Su S. Wang Z. Xu L. . (2025). The heterogeneity in psychological distress among undergraduate nursing students: a latent profile analysis. BMC Nurs. 24:801. doi: 10.1186/s12912-025-03373-6, 40598431 PMC12210687

[ref66] Wang C. L. Wang H. M. Li Y. Y. Dai J. Gu X. Q. Yu T. (2024). Factors influencing university students’ behavioral intention to use generative artificial intelligence: integrating the theory of planned behavior and AI literacy. Int. J. Hum. Comput. Interact. 41, 6649–6671. doi: 10.1080/10447318.2024.2383033

[ref67] Wang Q. Yan G. Hu Y. Ding G. Lai Y. (2024). Stress and emotion in a locked campus: the moderating effects of resilience and loneliness. Front. Psychol. 14:1168020. doi: 10.3389/fpsyg.2023.1168020, 38259567 PMC10800410

[ref68] Wei J. Ma Z. Eli B. (2025). Association of depressive symptom characteristics with sleep quality and psychological resilience in adolescents. Chin. J. Sch. Health 46, 842–846. doi: 10.16835/j.cnki.1000-9817.2025176

[ref69] Xie Z. Dai Z. Wei Y. Liu J. Zhang X. Zhong G. . (2024). The relationship between sleep profiles and anxiety and depression in addicted patients: a latent profile analysis. Sleep Med. 122, 192–197. doi: 10.1016/j.sleep.2024.08.005, 39186912

[ref70] Yang T. Huang H. (2003). An epidemiological study on stress among urban residents in social transition period. Zhonghua Liu Xing Bing Xue Za Zhi 9, 11–15. doi: 10.3760/j.issn:0254-6450.2003.09.004 14521764

[ref71] Yang Z. Peng H. Xin S. (2024). A longitudinal study on depression and anxiety among Chinese adolescents in the late phase of the COVID-19 pandemic: the trajectories, antecedents, and outcomes. Acta Psychol. Sin. 56, 482–496. doi: 10.3724/SP.J.1041.2024.00482

[ref72] Yang L. Zhu S. H. Zhang Y. Zhang J. F. Wang D. (2025). The configurational influence mechanism of risky behaviors among adolescents: a combined effect analysis of neuroticism, depression, anxiety, eating behavior, alcohol consumption, and quality of life. J. Affect. Disord. 397:120917. doi: 10.1016/j.jad.2025.120917 41419065

[ref73] Yeo G. C. Ong D. C. (2024). Associations between cognitive appraisals and emotions: a meta-analytic review. Psychol. Bull. 150, 1440–1471. doi: 10.1037/bul0000452, 39404856

[ref74] Yu T. Dai J. Wang C. L. (2023). Adoption of blended learning: Chinese university students’ perspectives. Humanit. Soc. Sci. Commun. 10:390. doi: 10.1057/s41599-023-01904-7

[ref75] Zhang Y. M. (1993). Psychiatric Rating Scale Manual. Changsha: Hunan Science and Technology Press, 35–42.

[ref76] Zhang J. Lau E. Y. Y. Hsiao J. H. W. (2019). Using emotion regulation strategies after sleep deprivation: ERP and behavioral findings. Cogn. Affect. Behav. Neurosci. 19, 283–295. doi: 10.3758/s13415-018-00667-y, 30460483

[ref77] Zhang J. Peng C. Chen C. (2024). Mental health and academic performance of college students: knowledge in the field of mental health, self-control, and learning in college. Acta Psychol. 248:104351. doi: 10.1016/j.actpsy.2024.104351, 38905949

[ref78] Zhang C. Zhao L. Dong T. Zhao J. Gao C. Zhao F. (2024). A prospective study of sleep status, anxiety, and depression levels of college students at a university in Shandong Province, China. Front. Psychol. 15:1361632. doi: 10.3389/fpsyg.2024.1361632, 38711753 PMC11070509

[ref79] Zhu K. Zhang Q. He B. Huang M. Lin R. Li H. (2022). Immersive virtual reality–based cognitive intervention for the improvement of cognitive function, depression, and perceived stress in older adults with mild cognitive impairment and mild dementia: pilot pre-post study. JMIR Serious Games 10:e32117. doi: 10.2196/32117, 35188466 PMC8902670

[ref80] Zung W. W. (1965). A self-rating depression scale. Arch. Gen. Psychiatry 12, 63–70. doi: 10.1001/archpsyc.1965.01720310065008, 14221692

[ref81] Zung W. W. (1971). A rating instrument for anxiety disorders. Psychosomatics 12, 371–379. doi: 10.1016/S0033-3182(71)71479-0, 5172928

